# Population Genomic Analysis Provides Insights Into the Evolution and Conservation of Two Critically Endangered Musk Deer Species

**DOI:** 10.1111/eva.70134

**Published:** 2025-07-31

**Authors:** Guotao Chen, Xiaonan Li, Yongxin Miao, Dapeng Pang, Hui Wang, Huizhong Fan, Baowei Zhang

**Affiliations:** ^1^ School of Life Sciences Anhui University Hefei China; ^2^ State Key Laboratory of Mycology, Institute of Microbiology Chinese Academy of Sciences Beijing China; ^3^ University of Chinese Academy of Sciences Beijing China; ^4^ CAS Key Laboratory of Animal Ecology and Conservation Biology, Institute of Zoology Chinese Academy of Sciences Beijing China

**Keywords:** adaptive evolution, deleterious mutations, demographic history, endangered, musk deer, population decline

## Abstract

Musk deer (*Moschus*), the sole genus in the family Moschidae, are critically endangered and face an uncertain future due to the limited understanding of their taxonomy, evolutionary history, genetic load, and adaptive evolution. These knowledge gaps hinder conservation efforts at crucial stages. Here, we conducted a comprehensive conservation genomic analysis by sequencing eight *M. anhuiensis* genomes and integrating public data from 15 *M. berezovskii* individuals. Phylogenomic and population genomic analyses confirmed that *M. anhuiensis* is a distinct phylogenetic species that diverged approximately 260 thousand years ago (kya). Both species experienced severe population bottlenecks, subsequently exhibiting marked genetic divergence. Over the past 200 kya, *M. berezovskii* has undergone multiple admixture events and bottlenecks, whereas *M. anhuiensis* has steadily declined and maintained a small, stable population. Anthropogenic activities have intensified these pressures, leading to sharp declines in both species. Notably, *M. anhuiensis* has accumulated homozygous deleterious mutations, thereby heightening its extinction risk. Moreover, selective sweep analysis revealed 32 positively selected genes, including olfactory receptor genes (*OLF3* and *OR6B1*), which are essential for foraging, reproduction, and social interactions; the proliferation‐related gene (*PDGFRA*), which responds to environmental changes and injury; and the thermoregulation gene (*CDH13*), which helps maintain body temperature stability in extreme conditions. These findings shed light on the speciation and evolutionary history of musk deer, offering crucial insights into their local adaptations and vulnerabilities. This work provides a foundation for targeted conservation efforts to avert extinction and safeguard biodiversity.

## 1 Introduction

Species misclassification results in suboptimal conservation decisions, particularly when managing endangered species with small, isolated populations (Gutiérrez and Helgen 2013; Zachos 2013). Such taxonomic misclassifications can substantially elevate the risk of species extinction. For instance, species such as the red wolf ( *Canis rufus* ) (Gese et al. 2015), the Asian elephant ( *Elephas maximus* ) (Fernando et al. 2001), and the Galápagos giant tortoises (Gaughran et al. 2023) have faced the threat of extinction due to prolonged taxonomic uncertainty, resulting in inadequate management and conservation efforts. To ensure effective conservation, the taxonomic status of certain endangered species has been reassessed, and extensive research has been conducted on their genetic diversity, mutation load, gene flow, inbreeding, and local adaptation. Notable examples include the red panda ( *Ailurus fulgens* ) (Hu et al. 2020), takin ( *Budorcas taxicolor* ) (Yang et al. 2022), and Eld's deer ( *Rucervus eldii* ) (Zheng et al. 2024). Therefore, research into species delimitation and their genetic backgrounds is essential for the successful conservation of endangered species (Yang et al. 2022).

With the development of sequencing technology, multiple high‐throughput sequencing methods have become widely used in conservation genomics. Genomic data derived from thousands to tens of thousands of genome‐wide markers enable comprehensive species and population delineation (Hu et al. 2017). This emerging field leverages whole‐genome sequencing (WGS) to assess species taxonomy and population dynamics, including hybridization events, population changes, and local genetic adaptations, with unprecedented resolution and accuracy (Zhao et al. 2013; Zhou et al. 2016; Hu et al. 2020; Yang et al. 2022). Critically, endangered species are elusive and often occur at low population densities, rendering large‐scale individual identification via multi‐locus approaches nearly unfeasible (Shafer et al. 2015). The results obtained by sequencing the genomes of a few individuals are comparable to those obtained by genotyping a large number of individuals using traditional markers, making WGS a powerful tool for conserving endangered species (Wright et al. 2020). Due to variations in population and species demographic histories (e.g., population fluctuations and founder effects), sensitivity to environmental changes, and life history traits, the extent of these processes differs across populations and species. Additionally, population fitness and survival may vary based on the proportion of rare large‐effect deleterious alleles and numerous small‐effect deleterious alleles in the founding individuals of these populations (Díez‐del‐Molino et al. 2018; Dussex et al. 2021; Hu et al. 2020). Currently, genomics studies have been widely used in many endangered species such as giant pandas (Zhao et al. 2013) red panda (Hu et al. 2020), snub‐nosed monkeys (Zhou et al. 2016), and kākāpō (Dussex et al. 2021). Thus, conservation genomics offers robust support for the development of targeted conservation strategies by enabling species delineation, population process reconstruction, inbreeding analysis, and deleterious mutation assessment.

Musk deer (*Moschus* spp.) are unique ruminants inhabiting forested and mountainous regions of Asia (Sheng et al. 1992). As the sole surviving member of the family Moschidae, musk deer face serious conservation challenges. These challenges primarily arise from illegal poaching, fueled by the high economic and medicinal value of musk, a sought‐after ingredient in luxury perfumes and traditional medicine (Yang et al. 2003; Yi et al. 2020; Liu et al. 2022). According to the International Union for Conservation of Nature Red List of Threatened Species v.2024 (https://www.iucnredlist.org), all musk deer species are classified as threatened, except for *Moschus moschiferus*, which is listed as vulnerable. Previous studies have identified six recognized musk deer species: (1) Forest musk deer ( *M. berezovskii* ), (2) Alpine musk deer ( *M. chrysogaster* ), (3) Black musk deer ( *M. fuscus* ), (4) Himalayan musk deer ( *M. leucogaster* ), (5) Siberian musk deer ( *M. moschiferus* ), and (6) Kashmir musk deer ( *M. cupreus* ).

Recently, the Anhui musk deer ( *M. anhuiensis* ) was identified as a potential seventh species; however, its taxonomic status remains debated. The Anhui musk deer was initially described in 1982 when Wang et al. identified a previously undocumented musk deer population in the Dabie Mountains. Following morphological comparisons, they classified it as a subspecies of the Siberian musk deer, designating it *Moschus moschiferus anhuensis* (Wang et al. 1982). This subspecies is geographically distinguished by its significant southern extension, reaching the central part of China, bordering the range of the forest musk deer. However, Groves and Feng (1986) and Wang et al. (1993) conducted morphological analyses including fur texture, coloration, body size, skull morphology, orbital shape, and habitat characteristics, concluding that the Anhui musk deer more closely resembles *M. berezovskii*. Subsequently, Su et al. (2001) confirmed that molecular evidence from mitochondrial genomes supports classifying the Anhui musk deer as a distinct species, rather than as a subspecies of *M. moschiferus* or *M. berezovskii* , as previously suggested by morphological studies. However, because mitochondrial markers capture only maternal lineages, they provide insufficient power to resolve recent speciation events or to infer detailed demographic histories. To date, no comprehensive whole‐genome study has simultaneously characterized taxonomy, genomic diversity, demographic history, and mutation load in this group (Camacho‐Sanchez et al. 2020), and such findings are essential for future assessments and conservation planning (Díez‐del‐Molino et al. 2018).

Here, we report the first whole‐genome re‐sequencing of eight wild *M. anhuiensis* individuals. We also analyzed publicly available genomes from 15 *M. berezovskii* and one *M. moschiferus* . Our objectives were to clarify the taxonomic status of *M. anhuiensis* and to assess genomic diversity, demographic history, mutation load, and adaptive evolution across musk deer. These findings are crucial to refine species classification and develop informed conservation strategies, ultimately supporting the preservation of these endangered ruminants.

## 2 Materials and Methods

### 2.1 Genome Resequencing, Reads Mapping and Variant Calling

**FIGURE 1:**
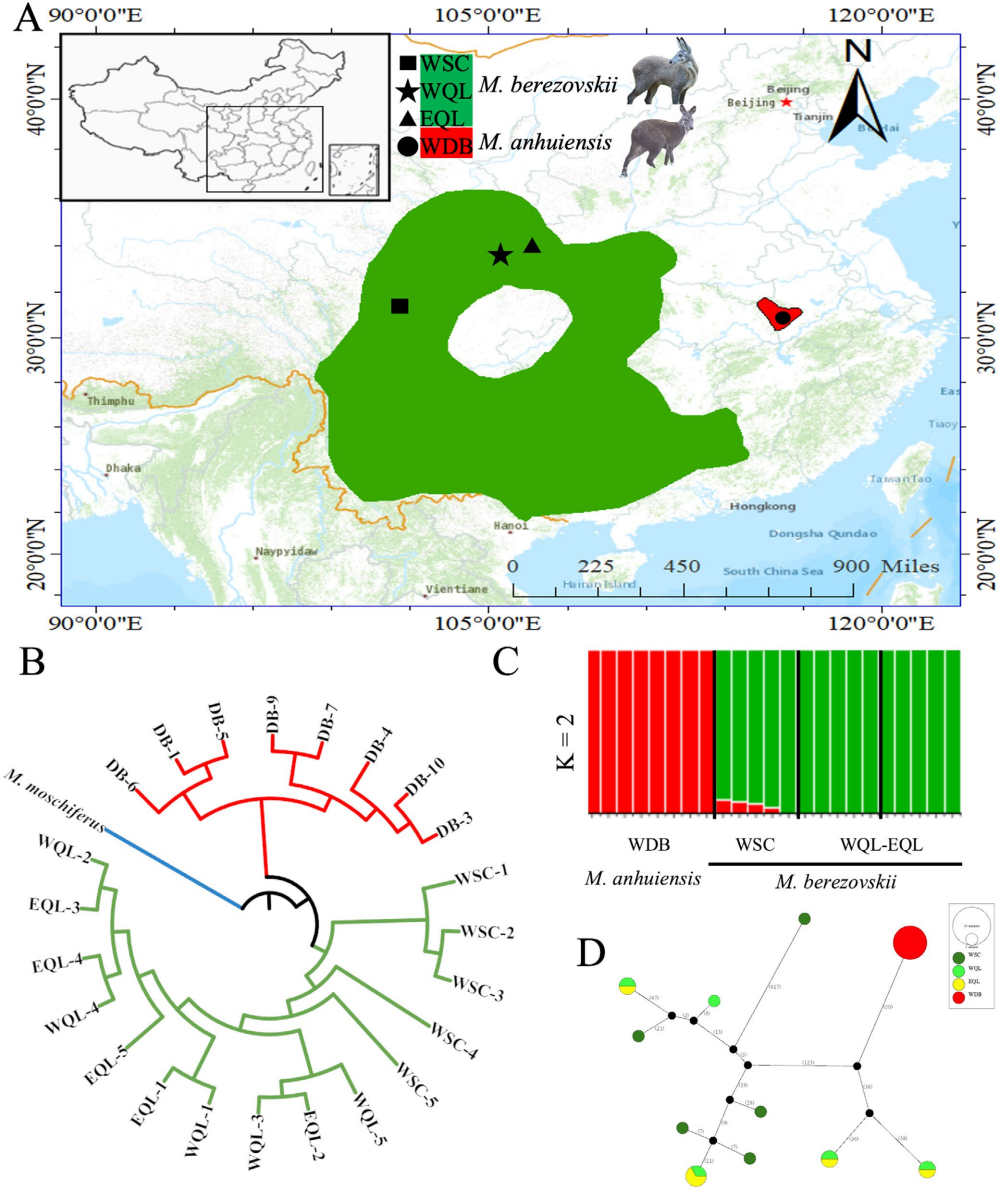
Population genetic structure based on autosomal single nucleotide polymorphisms (SNPs) and mitochondrial genomes of musk deer. (A) Geographic locations of wild musk deer samples under the background of the distribution range from the International Union for Conservation of Nature. (B) Neighbor‐joining tree based on genome‐wide SNP data. (C) ADMIXTURE analysis for *Moschus anhuiensis* and *M. berezovskii* , with the ancestry proportions of individuals shown for *K* = 2. (D) Network map based on mitochondrial genome haplotypes. EQL, captive *M. berezovskii* population from the east Qinling; WDB, wild population of *M. anhuiensis* from the Dabie Mountains; WSC, wild population of *M. berezovskii* from the west of Sichuan; WQL, captive population of *M. berezovskii* from the Qinling Mountains.

In this study, muscle tissue samples from eight *M. anhuiensis* individuals were collected from the Dabie Mountains (WDB) (Figure 1A). Genomic DNA was extracted using a standard phenol–chloroform protocol as previously described (Sambrook and Russell 2001). We prepared paired‐end libraries (300–500 bp inserts) and sequenced each sample to ~15× coverage on an Illumina HiSeq 2000 platform; the raw sequence data have been deposited in the Genome Sequence Archive National Genomics Data Center, China National Center for Bioinformation/Beijing Institute of Genomics, Chinese Academy of Sciences (BioProject RJCA029880). Additionally, resequencing data for *M. berezovskii* (*n* = 15) were obtained from the National Center for Biotechnology Information (NCBI) (BioProject accession numbers PRJNA765065 and PRJNA574937). These included five wild individuals from the western Sichuan population (WSC), five captive individuals from the western Qinling Mountains population (WQL), and five captive individuals from the eastern Qinling population (EQL), along with one *M. moschiferus* individual. Raw data were processed using FASTP v0.20.1 (Chen et al. 2018), which included trimming adapters, filtering low‐quality bases, and removing contaminating reads. The resulting clean reads were aligned to the genome *M. berezovskii* (reference genome: ls35.final.genome) (Fan, Li, et al. 2018) using the Burrows–Wheeler Aligner (BWA) v0.7.17‐r1188 (Li 2013), and the resulting BAM files were sorted using SAMtools v1.9 (Li et al. 2009). Variant calling was performed using the Genome Analysis Toolkit (GATK) v4.1.4.0 (McKenna et al. 2010). Before variant calling, a local realignment around indels and base quality score recalibration was performed using GATK tools to reduce false‐positive variant calls. GATK HaplotypeCaller was used to generate variant calls in the form of genomic variant call format (VCF) files. Next, the recommended GATK filtering parameters were used to filter the raw variant calls using the following set of parameters: QD < 2.0 || MQ < 40.0 || FS > 60.0 || SOR > 3.0 || MQRankSum < −12.5 || ReadPosRankSum < − 8.0. The resulting VCF file was further filtered using VCFtools v. 0.1.17 (Danecek et al. 2011) (parameters: ‐remove‐indels ‐‐max‐alleles 2 –minDP 4 ‐‐minQ 70). These methods were combined to generate final single nucleotide polymorphisms (SNPs).

### 2.2 Mitochondrial Genome Assembly

The mitochondrial genomes of all 23 musk deer individuals were assembled using GetOrganelle v1.7.4 (Jin et al. 2020). This was performed using WGS with the default parameters for assembling animal mitogenomes, including SPAdes k‐mer values of 21, 45, 65, 85, and 105, and a maximum of 10 extending rounds.

### 2.3 Phylogenetic Tree

Phylogenetic analysis was performed using the Neighbor‐Joining (NJ) algorithm to infer the evolutionary relationships among musk deer species. Separate NJ trees were generated using SNP data and mitochondrial genomes. Pairwise distances between individual pairs were computed using VCF2Dis v1.42 (https://github.com/BGI‐shenzhen/VCF2Dis). To evaluate tree robustness, 100 bootstrap replicates were generated as recommended by VCF2Dis to evaluate the robustness of the phylogenetic trees. Subsequently, a phylogenetic tree in the Newick format was constructed from these p‐distance matrices using FastME v2.0 (Lefort et al. 2015). The resulting tree was visualized using the Interactive Tree of Life (Letunic and Bork 2024), providing a detailed representation of evolutionary relationships among the three musk deer species.

### 2.4 Population Structure

To investigate population structure, two complementary approaches were employed: model‐based clustering analysis and principal component analysis (PCA). Model‐based clustering was conducted using ADMIXTURE v1.3.0 (Alexander et al. 2009), with the number of clusters (*K*) tested in the range of two to three. Cross‐validation error was minimized to determine the optimal *K* value. Additionally, PCA was performed using PLINK v1.90b6.8 (Purcell et al. 2007) to compute the top principal components, which were then visualized in a scatter plot.

### 2.5 Demographic History

#### 2.5.1 PSMC Analysis

To infer the demographic history of the three musk deer species, the Pairwise Sequentially Markovian Coalescent (PSMC) model (Li and Durbin 2011) was applied. High‐coverage sequencing data were obtained for each species, with a sequencing depth of 15× per individual. Reads were then mapped to the respective reference genome using the BWA. Low‐quality reads and duplicate sequences were removed using SAMtools. Next, demographic histories were inferred for each individual using PSMC with default parameters. Analyses were conducted assuming a generation time of 5 years and a mutation rate of 1.1 × 10^−8^ per site per generation (Liu et al. 2022).

#### 2.5.2 GONE Analysis

Given the recent rapid population decline, the extent of this reduction was assessed using GONE software, which estimates historical effective population size (*N*_e_) based on linkage disequilibrium (LD) across all available SNP pairs (Santiago et al. 2020). This method utilized LD patterns to estimate effective population size (*N*_e_) over recent generations, with the most reliable estimates corresponding to the past 0–200 generations. For input file preparation, filtered VCF files for each population were converted into MAP and PED formats using PLINK. 50 independent GONE replicates were executed using default parameters, including a maximum of 50,000 SNPs per analysis, a 0.05 hotspot threshold, and a maximum of 28 chromosomes.

### 2.6 Population Splits and Mixtures Analysis

Treemix v1.13 (Pickrell and Pritchard 2012) and Admixtools v2.08 (Maier et al. 2023) were employed to reconstruct the evolutionary history of *M. anhuiensis* and *M. berezovskii* populations. For Treemix analysis, SNPs in linkage disequilibrium (LD) were pruned using PLINK with the parameters (‐indep‐pairwise 50 5 0.2). Treemix was executed 20 times for each m value ranging from 1 to 3 (‐global ‐k 500 ‐se ‐bootstrap ‐noss), using *M. moschiferus* as the outgroup. The optimal migration parameter (*m* = 1) was identified using the OptM R package. Admixtools was applied with default parameters to generate hybrid plots and compute f3 outgroup statistics.

A multispecies coalescent‐based approach was also employed to estimate species divergence times, specifically using SNAPP (Bryant et al. 2012) within the BEAST2 v2.4.8 (Bouckaert et al. 2014) to infer the species tree. Three representative samples were selected per species or population. To optimize computational efficiency, 2000 biallelic SNPs were randomly sampled. A total of 100,000 iterations were executed, with sampling occurring every 50 generations. The SNAPP‐inferred species tree was visualized using DensiTree v2.2.6 (Bouckaert 2010).

### 2.7 Within‐Population/Species Statistics

The filtered VCF dataset was stratified by geographic population to compute within‐population genetic diversity metrics. Nucleotide diversity (*π*) was estimated genome‐wide in 10 kb nonoverlapping windows using Pixy v1.2.7.beta1 (Korunes and Samuk 2021). Since Pixy emphasizes the necessity of incorporating missing data, including invariant sites, SNP calling was conducted to include invariant sites required by Pixy. Variants were filtered using VCFtools to exclude indels, sites with sequencing depths below 5, and loci with more than 20% missing data (‐‐remove‐indels ‐‐minQ30 ‐‐minDP 5 ‐‐max‐missing 0.8). Separate VCF files were generated for invariant and variant sites using VCFtools (‐‐max‐maf 0 and ‐‐mac 1) following the Pixy tutorial. Individuals with greater than 80% missing data, sites with excess observed heterozygosity (> 0.6), and variants in linkage disequilibrium (LD) were removed from the variant site VCF, following previously described methods.

Observed heterozygosity per individual was calculated using the “‐‐het” function in VCFtools. Runs of homozygosity (ROH) were identified using parameters optimized for low‐density data (Ceballos et al. 2018): ‐‐homozyg‐snp 50, ‐‐homozyg‐kb 300, ‐‐homozyg‐density 50, ‐‐homozyg‐gap 1000, ‐‐homozyg‐window‐snp 50, ‐‐homozyg‐window‐het 2, ‐‐homozyg‐window‐missing 5, and ‐‐homozyg‐window‐threshold 0.05.

### 2.8 Linkage Disequilibrium Decay

To examine linkage disequilibrium (LD) decay in the studied species, PopLDdecay v3.43 software was employed (Zhang et al. 2019). Initially, genetic variants were filtered to retain SNPs with MAF > 0.05 and missingness < 10%. Next, PopLDdecay was applied with default parameters to compute pairwise LD measures (*r* ^2^) between genetic variants across the genome. Subsequently, the average LD decay curves were computed and visualized across distance bins using R.

### 2.9 Mutational Load Estimation

To predict the functional effects of the variants, Sorting Intolerant From Tolerant 4G (SIFT_4G) (Vaser et al. 2016) was used to annotate the SNP dataset. A musk deer‐specific database was constructed using UniRef90 (https://www.uniprot.org/, accessed March 2022) as the reference protein set. The *M. berezovskii* genome annotation (ls35.final.genome) was obtained from the ZOODNA database. The musk deer SIFT_4G database was generated using SIFT4G_Create_Genomic_DB within the SIFT_4G framework. SIFT scores ranged from 0 to 1, with non‐synonymous variants assigned a score < 0.05 classified as putatively deleterious. Deleterious variants were predicted using ancestral alleles rather than reference sequences to reduce reference bias. The probabilities of ancestral and derived allelic states were estimated using maximum likelihood with est‐sfs (Keightley and Jackson 2018). Mutational load for each variant type was computed using an additive model: (2 × homozygous variants + number of heterozygous variants) (Henn et al. 2015).

Next, variant frequency differences across impact categories between *M. anhuiensis* and *M. berezovskii* were estimated using a method adapted from Xue et al. (2015). A population comparison statistic was then computed to quantify derived allele distributions at sites unique to each species. For each category of variants, the observed allele frequency in population *x* at site *i* was estimated as $f_{i}^{x}=d_{i}^{x}/n_{i}^{x}$, where $n_{i}^{x}$ represents the total number of called alleles in population *x*, and $d_{i}^{x}$ denotes the total count of derived alleles. Similarly, $f_{i}^{y}$ was defined for population *y*. Then, for each C category of variants we estimated.

$${\text{Freq}}_{\mathrm{pop}-x}\left(C\right)=\sum_{\mathrm{i\epsilon C}}f_{i}^{x}\left(1-f_{i}^{y}\right)$$

Finally, the frequency ratio (Freq_pop‐*x*_/Freq_pop‐*y*_) was computed, where a value of 1 indicates no change in frequency, *R*_*xy*_ > 1 signifies a decrease in frequency in population *y* relative to population *x*, and *R*_*xy*_ < 1 denotes an increase in frequency in population *y* relative to population *x*.

### 2.10 Identification of Selection Signatures

Two complementary methods were utilized to identify signatures of selection across populations: XP‐CLR v1.1.2 (Cross‐Population Composite Likelihood Ratio Test) (Chen et al. 2010) and XP‐EHH v2.1 (Cross‐Population Extended Haplotype Homozygosity) (Szpiech and Hernandez 2014). XP‐CLR, a composite likelihood ratio‐based approach, identifies selection signatures by analyzing allele frequency differences between populations. This approach is particularly effective in cases where populations are small and exhibit low genetic variation. Conversely, XP‐EHH assesses the extent of extended haplotype homozygosity between populations, leveraging this measure to detect genomic regions subjected to long‐term selection. Whereas XP‐CLR focuses on allele frequency differences among populations, XP‐EHH is particularly sensitive to regions where extended haplotypes have reached fixation or near fixation.

For selection analysis, XP‐CLR was applied using a 100 kb sliding window, and XP‐EHH analyses were performed across populations using a 50 kb nonoverlapping window. Genomic regions within the top 1% of significance in both methods were designated as candidate regions for further analysis. Genes within these candidate regions were identified based on the gff3 annotation file. In summary, genomic regions identified by both methods were designated as candidate regions, and Tajima's *D* and fixation index (FST) were computed using VCFtools to integrate the results.

### 2.11 Variation Annotation

Functional effects of identified SNPs were predicted using SnpEff v4.3t (Cingolani et al. 2012) with default parameters. Variant effects were categorized as synonymous, missense, splice‐site, stop‐gain, and frameshift.

## 3 Results

### 3.1 Genomic Evidence of Two Phylogenetic Species in Musk Deer

Whole‐genome resequencing data were generated for eight *M. anhuiensis* individuals, and publicly available genomes of *M. berezovskii* (*n* = 15) and *M. moschiferus* (*n* = 1) were included as outgroups (Figure 1A, Table S1). This analysis employed a 2.8 Gb reference genome of *M. berezovskii* , yielding an average sequencing depth of ~15× per individual and 99.8% genome coverage. The single nucleotide polymorphism (SNP)‐calling strategy implemented by GATK identified 53,724,673 autosomal SNPs for further analysis. Analyses including genome‐wide SNPs, phylogenetic trees, ADMIXTURE, and PCA revealed pronounced genetic differentiation (Figure 1B,C, Figures S1 and S2). The *M. anhuiensis* population in the Dabie Mountains diverged markedly from the *M. berezovskii* population (Figure 1A,B). Mitochondrial genome comparisons also supported substantial divergence between the two species (Figure 1D). Haplotype network analysis of mitochondrial genomes from 23 individuals revealed that *M. anhuiensis* exhibited no haplotype variation, with only a single mitochondrial haplotype detected, whereas the NJ tree of mitochondrial DNA highlighted the substantial genetic divergence between the two species (Figure 1D, Figure S3). Collectively, nuclear and mitochondrial data provide robust support for species‐level differentiation between *M. anhuiensis* and *M. berezovskii*. In addition to genetic differentiation, significant morphological differences exist between the *M. anhuiensis* and *M. berezovskii* . Morphologically, *M. anhuiensis* was reported (Li et al. 1999) to have a narrower zygomatic width, longer rostrum, and lacrimal bone lengths in skull morphology. It is also slightly larger in body size, with grayish‐brown fur, distinct neck stripes, and body spots, which are either lacking or highly variable in *M. berezovskii*. These findings support the recognition of *M. anhuiensis* and *M. berezovskii* as distinct evolutionary lineages within the musk deer family.

### 3.2 *Moschus anhuiensis* and *M. berezovskii* Exhibit Distinct Evolutionary Processes

**FIGURE 2:**
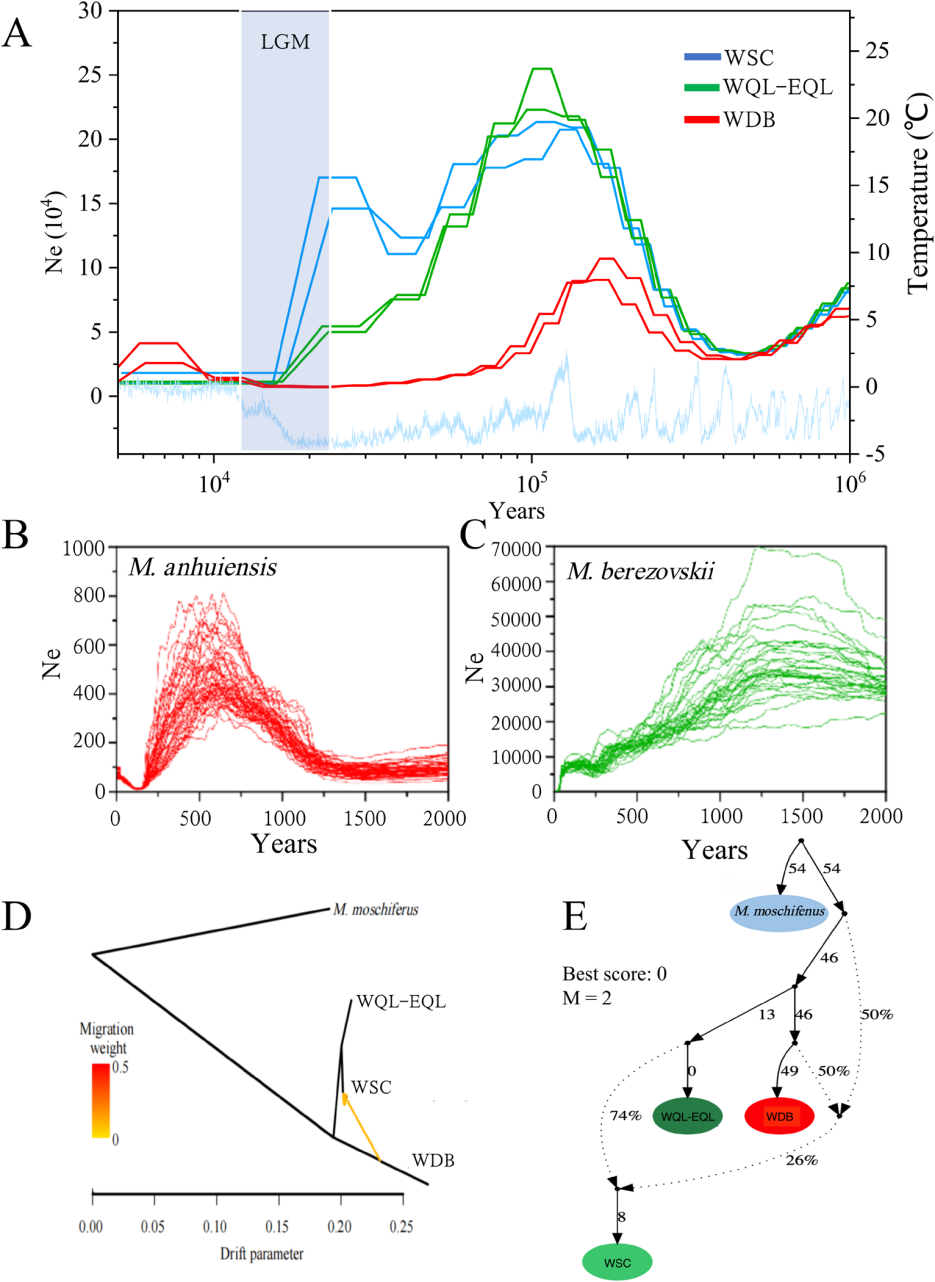
Demographic history of *Moschus anhuiensis* and *M. berezovskii*. (A) Inferred from autosomal DNA using the PSMC model. The fluctuations in effective population size (*N*_e_) from 5 thousand years ago (kya) to 1 million years ago (mya) are estimated based on the 5‐year generation time and 1.1 × 10^−8^ per site per generation mutation rate assumptions. The red line represents the estimated *N*_e_ of *M. anhuiensis* (WDB), whereas the green and blue lines represent the WQL‐EQL and WSC populations of *M. berezovskii* , respectively. The light blue dashed line represents the average global temperature over the past one million years. The blue shading represents the Last Glacial Maximum (LGM: ~21 kya). (B, C) GONE analysis of *N*_e_ for *M. anhuiensis* and *M. berezovskii* over the past 2000 years. (D) Gene flow analysis for *M. anhuiensis* and *M. berezovskii*. (E) Admixtools analysis for *M. anhuiensis* and *M. berezovskii* , where the point of convergence of the two dashed lines represents a single admixture event, and the values adjacent to the dashed lines indicate the proportion of the admixture.

Understanding the demographic history and gene flow between *M. anhuiensis* and *M. berezovskii* is essential for assessing their population structure, evolutionary trajectories, and conservation needs. We reconstructed demographic history using the Pairwise Sequentially Markovian Coalescent (PSMC) and GONE models, whereas TreeMix and Admixtools analyses were conducted to evaluate gene flow between species and their geographic populations. PSMC results showed a shared demographic trajectory between *M. anhuiensis* and *M. berezovskii* until ~400 thousand years ago (kya) (Figure 2A). After an ancestral bottleneck, the two lineages began to diverge. SNAPP analysis dated the divergence between *M. anhuiensis* and *M. berezovskii* to ~260 kya (Figure S4). *M. anhuiensis* experienced population growth and peaked ~200 kya, before being followed by a prolonged population decline. In contrast, *M. berezovskii* continued to expand, reaching its maximum effective population size (*N*_e_) ~100 kya before contracting (Figure 2A). Notably, *M. berezovskii* underwent a pronounced population expansion ~40 kya, followed by a sharp contraction and a severe bottleneck coinciding with the Last Glacial Maximum. This bottleneck was detected in two geographic populations of *M. berezovskii* in this study (Figure 2A). Meanwhile, *M. anhuiensis* maintained a relatively stable, small *N*_e_ during this period, with only a minor expansion after the glacial period, followed by a contraction. To assess recent anthropogenic effects, we reconstructed population trends over the past 2000 years. GONE analysis revealed a sharp decline in *N*_e_ over the past ~2000 years. The peak *N*_e_ of *M. anhuiensis* remained below 800 throughout the period examined (Figure 2B), which was significantly lower than the maximum *N*_e_ of *M. berezovskii* (~30 kya) (Figure 2C). However, both species have undergone severe population declines in recent history, with *M. anhuiensis* currently at critically low effective population levels (Figure 2B,C).

We further explored gene flow between *M. anhuiensis* and wild *M. berezovskii* populations (WSC and WQL‐EQL, respectively) using TreeMix and Admixtools. TreeMix analysis detected a gene flow signal between the two species, specifically from *M. anhuiensis* into the WSC population of *M. berezovskii* (Figure 2D). This suggests historical genetic exchange between these populations, despite their divergence. To further investigate the admixture history, Admixtools was used, which revealed that the WSC population of *M. berezovskii* experienced two distinct episodes of admixture. The first one occurred with ancestors of the *M. anhuiensis* population and an unknown musk deer source, contributing to approximately 50% of the genetic material. The second admixture event involved gene flow from the WQL‐EQL population of *M. berezovskii* , resulting in the current WSC population with a predominantly WQL‐EQL genetic background (74%) (Figure 2E).

### 3.3 *Moschus anhuiensis* Displayed Lower Genetic Diversity and Severe Inbreeding Depression

**FIGURE 3:**
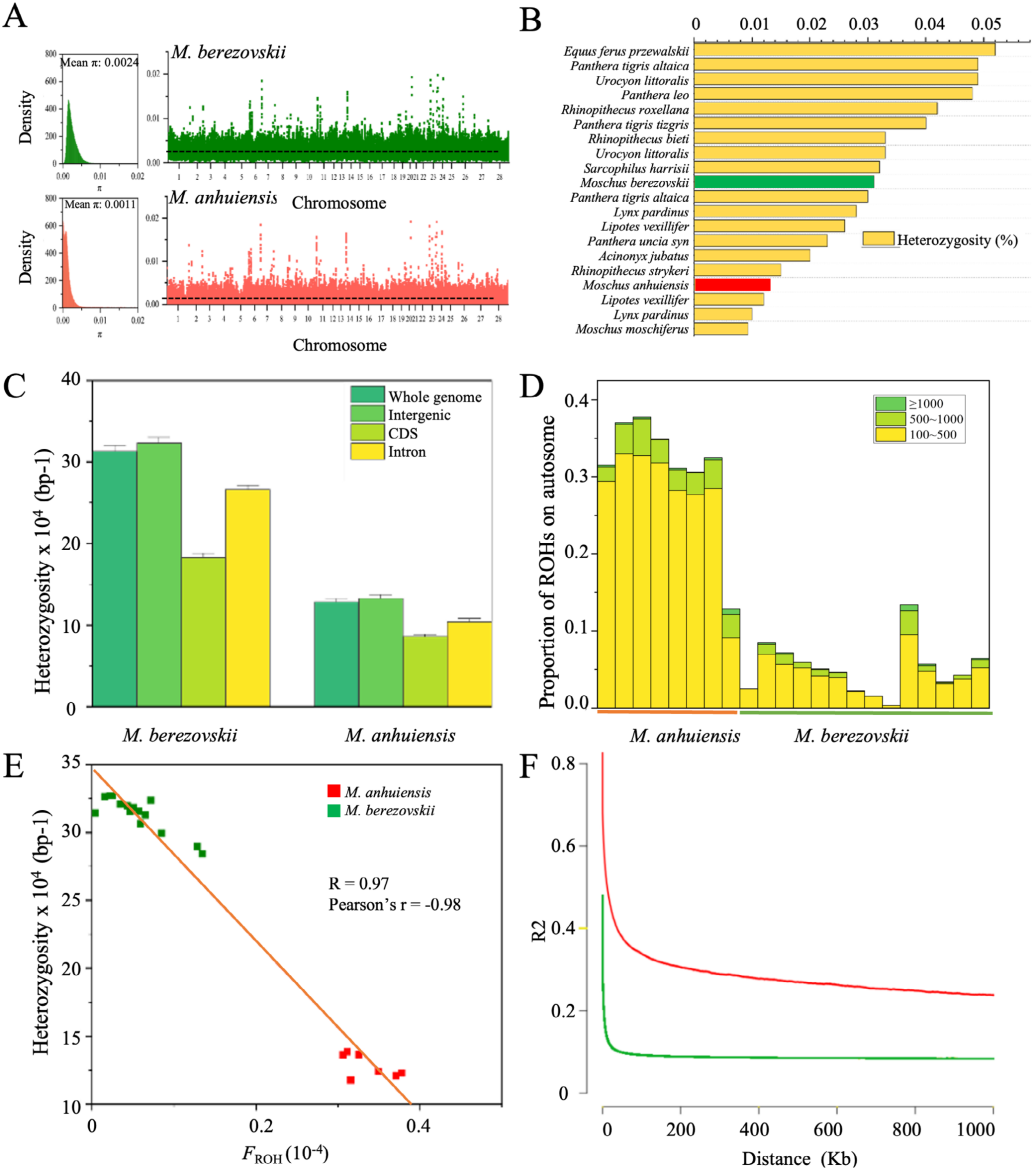
Genomic variation, inbreeding, and linkage disequilibrium of two musk deer species. (A) Nucleotide diversity across chromosomes is calculated for 10 kb nonoverlapping windows. (B) Genome heterozygosity of two musk deer compared to those of other endangered species (Hu et al. 2020). (C) The heterozygosity density in different genomic regions (whole genome, intergenic, coding sequence [CDS], and intron) of each individual. (D) The proportion of runs of homozygosity (ROHs) regions in different length categories (100–500, 500–1000, and > 1000 kb) across the autosomes. (E) The correlation between the proportion of the genome in runs of homozygosity (*F*_ROH_) and heterozygosity. (F) Linkage disequilibrium of *Moschus anhuiensis* and *M. berezovskii* .

Understanding genomic diversity, inbreeding levels, and linkage disequilibrium (LD) is crucial for assessing the health and long‐term viability of small populations. In this study, we compared genome‐wide autosomal genetic diversity (*π*) between *M. anhuiensis* and *M. berezovskii* . *M. anhuiensis* exhibited markedly lower genetic diversity (*π* = 0.0011) than *M. berezovskii* (*π* = 0.0024) (Figure 3A). Analysis of genetic diversity distribution across 50 kb nonoverlapping windows in the autosomal genome revealed that *M. anhuiensis* consistently displayed lower diversity than *M. berezovskii* , with certain regions showing substantial declines (Figure 3A). The genome‐wide autosomal heterozygosity of *M. anhuiensis* (0.013%) was substantially lower than that of *M. berezovskii* (0.031%) and other endangered mammals (Figure 3B). Due to the small effective population size (*N*_e_) and historical bottleneck events in *M. anhuiensis* , we assessed genome‐wide heterozygosity and the genomic fraction within runs of homozygosity (*F*_ROH_). Specifically, coding regions exhibited the lowest heterozygosity in both species, whereas intergenic regions showed the highest heterozygosity (Figure 3C). Next, runs of homozygosity (ROHs) were extracted from each individual and categorized into three length classes. On average, *M. anhuiensis* exhibited 4190 ± 577 ROHs, accounting for 33.6% ± 0.029% of the genome, compared to only 600 ± 318 ROHs (5.6% ± 3.6% of the genome) in *M. berezovskii* . A substantial proportion of ROHs in *M. anhuiensis* were short (100–500 kb) (Figure 3D). The high proportion of ROHs underscores severe inbreeding in *M. anhuiensis* , contributing to further reductions in genetic diversity. Correlation analysis revealed a strong inverse relationship between individual heterozygosity and *F*_ROH_, with *M. berezovskii* and *M. anhuiensis* forming distinct clusters (*R* = 0.97, Pearson's *r* = −0.98) (Figure 3E), which further supports the notion that inbreeding in these genetically distinct species has led to varying degrees of genetic diversity loss. Finally, LD analysis revealed a slower LD decay rate in *M. anhuiensis* compared to *M. berezovskii* (Figure 3F), indicating that the bottleneck caused by population decline has resulted in decreased genomic diversity and increased inbreeding.

### 3.4 *Moschus anhuiensis* Has a Relatively Higher Deleterious Mutations Load

**FIGURE 4:**
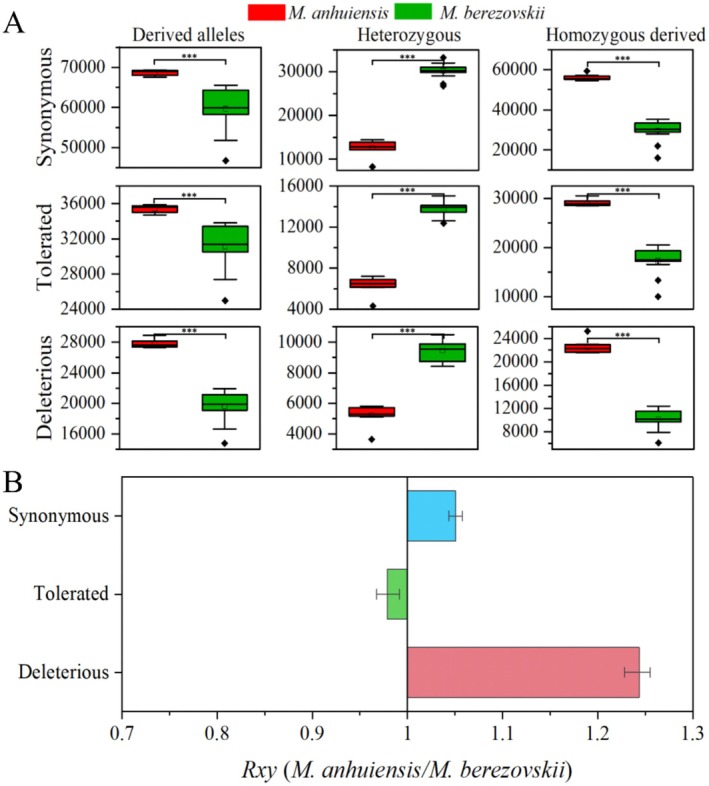
Comparison of deleterious genetic variation in *Moschus anhuiensis* and *M. berezovskii*. (A) The plot illustrates the distribution of derived alleles per individual for autosomal variants. The total count of derived alleles is determined by counting each heterozygous genotype once and each homozygous‐derived genotype twice. Only autosomal variants are considered in this analysis. Statistical significance was assessed using Welch's two‐sample *t*‐test (***p < 0.001). (B) *R*_*xy*_ ratio of derived alleles for synonymous, tolerated, and deleterious variants. *R*_*xy*_ < 1 indicates a relative frequency deficit of the corresponding category in *M. berezovskii* compared to that of *M. anhuiensis* . Whiskers represent a 95% confidence interval (CI).

Inbreeding can lead to an increase in homozygosity for deleterious mutations, thereby affecting the fitness and survival of the species. To further assess the genetic load in the *M. anhuiensis* population, we annotated mutations in coding regions and assessed their functional consequences. Using *M. moschiferus* as an outgroup, the alleles were polarized as either ancestral or derived (Figure 4A). Next, we estimated the proportions of heterozygous mutations, synonymous SNPs, tolerated non‐synonymous SNPs (tSNPs), and deleterious mutations (DEL) across all samples (Figure 4A). This analysis revealed that *M. anhuiensis* harbors a significantly higher additive genetic load than *M. berezovskii* . Specifically, homozygous variants were significantly more prevalent in *M. anhuiensis* than in *M. berezovskii* (*p* < 0.05, Mann–Whitney *U* test), whereas heterozygous variants were less common in *M. anhuiensis* . These results suggest that deleterious alleles have become homozygous in *M. anhuiensis*, likely due to strong genetic drift, which weakened the efficiency of the purifying selection. Notably, the average number of deleterious homozygous mutations in *M. anhuiensis* was 122.2% higher than that in *M. berezovskii* (*p* < 0.05) (Figure 4A), highlighting the substantial burden of deleterious homozygous mutations in *M. anhuiensis* . Additionally, the average number of homozygous synonymous mutations was 90.0% higher, and the average number of tSNPs was 68.0% higher in *M. anhuiensis* compared to *M. berezovskii* . These findings indicate that the accumulation of homozygous DEL variants contributed more significantly to the genetic load of *M. anhuiensis* . The ratio of derived alleles (*R*_*xy*_) statistic further illustrated this pattern, with *M. anhuiensis* exhibiting a higher proportion of DEL (*R*_*xy*_ = 1.24) compared to *M. berezovskii* (Figure 4B). Collectively, these findings indicate that inbreeding and drift have elevated genetic load in *M. anhuiensis* , potentially compromising its fitness.

### 3.5 Genomic Signatures for Selection and Local Adaptation

**FIGURE 5:**
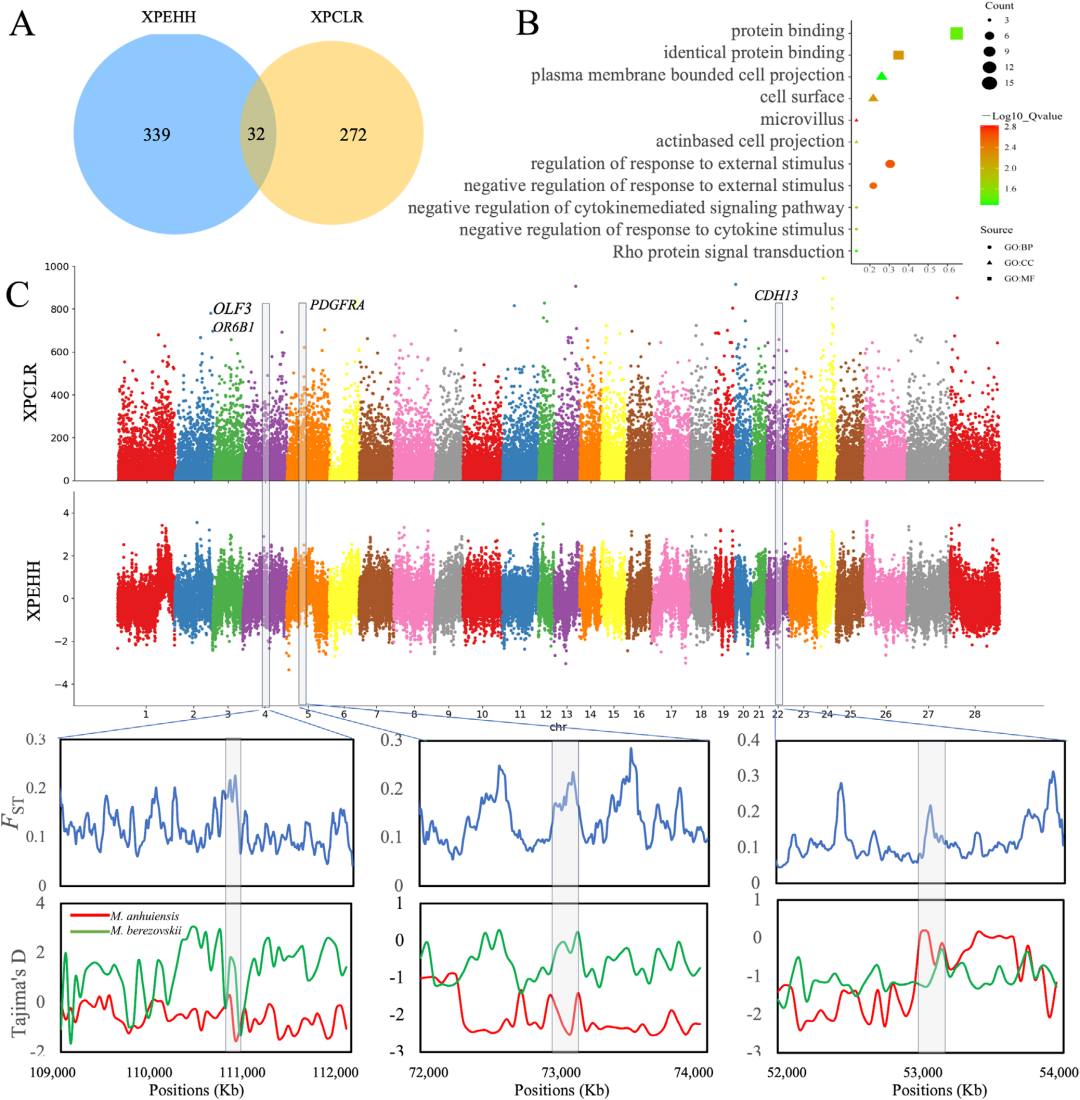
Genome‐wide selective sweep of *Moschus anhuiensis* and *M. berezovskii* . (A) Venn diagram illustrating the overlap between two statistical methods (XP‐EHH and XP‐CLR). (B) Genes identified within the regions of selective sweep, which were utilized for enrichment analysis. (C) Genome‐wide adaptation signals for *M. anhuiensis* , as detected by XP‐CLR and XP‐EHH, along with plots of *F*_ST_ and Tajima's *D* values in regions surrounding the *OLF3*, *OR6B1*, *PDGFRA*, and *CDH13* genes.

Given their divergent geographic distributions and environmental conditions occupied by the two musk deer species, as well as their prolonged genetic divergence, we analyzed the genomic features associated with selection and local adaptation in these species. XP‐CLR and XP‐EHH scans were used to detect genomic regions under selection, resulting in 272 and 339 genes, respectively. Among these, with 32 genes jointly identified by both methods (Figure 5A, Table S2). A functional enrichment analysis revealed that some of these genes are involved in pathways such as “regulation of response to external stimulus” (GO:0032101, *p* = 0.0025) and “negative regulation of response to external stimulus” (GO:0032102, *p* = 0). Furthermore, the analysis identified “negative regulation of cytokine‐mediated signaling pathway” (GO:0001960, *p* = 0.013) and “negative regulation of response to cytokine stimulus” (GO:0060761, *p* = 0.014) as functional categories with potential relevance (Figure 5B, Table S3). These genes may play a role in the environmental adaptation of *M. anhuiensis* in the Dabie Mountains.

Among the commonly identified selection regions, several noteworthy genes merit particular attention. Two genes from the olfactory receptor family (*OLF3* and *OR6B1*) encode olfactory receptor proteins that interact with odor molecules in the nasal passages, initiating neuronal responses and triggering scent perception. The *PDGFRA* gene (platelet‐derived growth factor receptor) encodes a catalytic receptor with intracellular tyrosine kinase activity, regulating numerous biological processes, including embryonic development, angiogenesis, cell proliferation, and differentiation. The *CDH13* gene encodes a calcium‐dependent cell adhesion protein, cadherin, implicated in synaptic regulation and neuronal connectivity. Additional confirmation of these selected genes was achieved through *F*_ST_ and Tajima's *D* analysis (Figure 5C). In sum, these genes appear to play a pivotal role in the local adaptation and evolutionary history of *M. anhuiensis* .

## 4 Discussion

Our population genomic analyses demonstrate that *M. anhuiensis* is a genetically distinct species that diverged from *M. berezovskii* ~260 kya. Both species have undergone severe population bottlenecks, leading to significant differentiation. In the past 200 kya, *M. berezovskii* has undergone multiple population admixture events and bottlenecks, whereas *M. anhuiensis* has experienced a continuous decline, maintaining a small but stable population. Human activities have intensified these pressures, leading to marked declines in the populations of both musk deer species. Notably, *M. anhuiensis* has accumulated deleterious homozygous mutations, increasing its susceptibility to extinction.

### 4.1 Genomic Evidence for Species Differentiation of *M. anhuiensis* and *M. berezovskii*

Early morphological studies based on fur texture, body color, body size, skull dimensions, orbital shape, and habitat characteristics prompted Groves and Feng (1986) and Wang et al. (1993) to propose classifying *M. anhuiensis* as a subspecies of *M. berezovskii* rather than as a subspecies of *M. moschiferus* . However, the results of our comprehensive genomic analysis, which integrated whole‐genome SNP data and mitochondrial genome sequences, challenge this subspecies hypothesis. Specifically, phylogenetic analyses reveal substantial genetic divergence between *M. anhuiensis* and *M. berezovskii* , with an estimated divergence time of approximately 260,000 years, whereas *M. moschiferus* diverged earlier. Mitochondrial and nuclear genomic data jointly support the classification of *M. anhuiensis* as a distinct evolutionary lineage. These genomic findings, together with previously reported morphological differences (Li et al. 1999; Su et al. 2001), robustly support the recognition of *M. anhuiensis* as an independent species. Moreover, our geographic analyses suggest that natural barriers, such as the Yangtze and Han Rivers, have been pivotal in facilitating the divergence of these lineages. Taken together, these data refute the earlier subspecies classification. These results underscore the need for species‐specific conservation strategies, which are critical for ensuring the survival of these endangered populations.

### 4.2 *Moschus berezovskii* and *M. anhuiensis* Experience Different Demographic Histories

Extensive population genomic analyses conducted on *M. anhuiensis* confined to the Dabie Mountains reveal that its population is both small and isolated. In recent decades, this trend has been exacerbated by anthropogenic pressures, including habitat destruction and poaching (Yang et al. 2003; Zhou et al. 2004). Relative to *M. berezovskii* , *M. anhuiensis* exhibited markedly lower genetic diversity and a higher degree of inbreeding, as evidenced by elevated *F*_ROH_ values and a greater proportion of deleterious homozygous mutations. These genetic factors, compounded by recent human pressures, have propelled this species to the brink of extinction. Demographic analyses further indicate that the evolutionary histories of *M. anhuiensis* and *M. berezovskii* differ significantly. Our findings suggest that *M. anhuiensis* and *M. berezovskii* likely diverged from a common ancestor ~260 kya. During subsequent population expansion, a subpopulation likely dispersed into the Dabie Mountains, where they evolved into *M. anhuiensis* . The Dabie Mountains, serving as a refugium during glacial periods, enabled *M. anhuiensis* to persist through multiple glaciations while maintaining a relatively small yet stable population. Similarly, the region's *Pachyhynobius shangchengensis* (Zhao et al. 2013) successfully navigated several glacial cycles. However, over the past 350 years, the population size of *M. anhuiensis* has declined as China's human population has steadily grown (Su et al. 2001). Excessive deforestation for urban development and the construction of infrastructure such as towns, roads, and railways has resulted in severe habitat loss and a reduction in the effective population size of *M. anhuiensis*. Moreover, the high commercial value of its pelts and musk has rendered it a frequent target of poaching, further exacerbating its survival crisis.

### 4.3 Inbreeding Impact for *M. anhuiensis*

Genetic analyses indicate that inbreeding significantly impacts *M. anhuiensis* , as evidenced by reduced heterozygosity and ROHs across its genome. Notably, the extent of ROHs in *M. anhuiensis* is considerably greater than that observed in *M. berezovskii* , suggesting a higher level of inbreeding. Furthermore, the markedly elevated LD in *M. anhuiensis* further underscores the severe genetic pressures confronting this species (Ceballos et al. 2018). The accumulation of deleterious mutations, particularly homozygous missense alleles, likely contributes to the increased genetic vulnerability of *M. anhuiensis*. Mutation load, an important indicator of genetic risk in small populations (von Seth et al. 2021), not only compromises individual fitness but also limits the species' adaptive potential in fluctuating environments. In small populations, genetic drift can fix these deleterious alleles, thereby exacerbating mutation load and impeding adaptive evolution (Lynch et al. 1995; Hedrick 2009). These findings emphasize the urgent need for conservation programs that prioritize genetic management. Although *M. berezovskii* exhibits lower inbreeding levels, the presence of deleterious mutations in *M. anhuiensis* poses an immediate threat to its long‐term viability. The shared mitochondrial haplotype among all *M. anhuiensis* individuals suggests a history of severe genetic drift or a historical bottleneck that reduced genetic diversity. This genetic uniformity may heighten the population's susceptibility to environmental changes, diminish its adaptive capacity, and accelerate the accumulation of deleterious mutations, thereby compromising its health and long‐term survival. Overall, these results underscore the critical need for targeted conservation strategies aimed at mitigating genetic load and promoting population recovery. Despite the robustness of our findings, certain limitations remain. The restricted sample size may constrain the broader applicability of the findings, and difficulties in obtaining samples from certain geographic regions might result in an underrepresentation of genetic variation. Future research should therefore expand the sampling scope and incorporate additional environmental and ecological data to more comprehensively validate these results and mitigate potential biases arising from data or methodological limitations.

### 4.4 Local Adaptive Evolution Despite Long‐Term Bottlenecks

Despite facing severe survival threats, the *M. anhuiensis* population exhibits clear signatures of adaptive evolution. Our analyses identified several candidate genes associated with olfaction, reproduction, and temperature adaptation, each enriched in relevant Gene Ontology categories. Notably, the olfactory receptor genes *OLF3* and *OR6B1*, involved in chemosensory perception, have been associated with environmental adaptation in other species. In *M. anhuiensis* , selection on olfactory receptor genes is likely driven by the need for effective communication in the fragmented, low‐density habitats of the Dabie Mountains, where enhanced scent detection aids in territorial marking and mate attraction (Fan, Zhang, et al. 2018; Fan, Li, et al. 2018; Li et al. 2018). The olfactory receptor gene *OR6B1* has been identified as a key environmental adaptation gene in gray wolves (Schweizer et al. 2016) and has been linked to food preference in human genome‐wide association studies (Cole et al. 2020). Additionally, the *PDGFRA* gene, which is crucial for cellular proliferation and temperature adaptation, is also under selection in *M. anhuiensis* . The harsh climatic conditions of the Dabie Mountains, marked by extreme temperature fluctuations, may have favored variants of *PDGFRA* that confer improved cellular resilience and thermoregulation. Evidence from studies on high‐altitude cattle breeds (Zinovieva et al. 2020) and Markhoz goats (Nazari‐Ghadikolaei et al. 2018) supports the notion that *PDGFRA* is responsive to environmental thermal stress, and it may also influence phenotypic traits such as coat color differences observed between *M. anhuiensis* and *M. berezovskii* . In addition, our identification of the *CDH13* gene, which encodes a member of the cadherin superfamily, suggests that resistance to oxidative stress and vascular protection are important for local adaptation. The protective role of *CDH13* against apoptosis and its association with blood pressure regulation in indigenous populations (Deng et al. 2014; Ivanov et al. 2004) further indicate its potential adaptive significance in the context of the environmental challenges encountered by *M. anhuiensis* . Overall, our findings suggest that prolonged isolation of *M. anhuiensis* from its *M. berezovskii* relatives has driven local adaptations in response to the distinct ecological pressures of the Dabie Mountains. These pressures include extreme temperature fluctuations, habitat fragmentation, altered resource availability, and social structure changes that necessitate enhanced olfactory communication. These selective pressures have promoted the fixation of adaptive alleles in key genes, thus facilitating the survival of the species in its unique environment.

### 4.5 Conservation Strategies for *M. anhuiensis* and *M. berezovskii*

For millennia, musk deer have faced intense overhunting due to the high commercial and medicinal value of musk, a key ingredient in perfumes and traditional medicine. Consequently, musk deer populations are experiencing a rapid decline and face an imminent risk of extinction. Misclassification of fundamental taxa can result in misinterpretation of evolutionary history, misidentification of adaptive mechanisms, and flawed conservation strategies for threatened species (Gutiérrez and Helgen 2013; Zachos 2013). Our findings support recognizing *M. anhuiensis* and *M. berezovskii* as distinct species and treating them as independent conservation management units. To achieve this, we propose the following conservation measures: (1) Establish protected areas and ecological corridors to safeguard habitats, minimize human disturbances, and facilitate gene flow. (2) Strengthen legal protections by strictly prohibiting hunting and illegal trade while enhancing enforcement and monitoring within conservation zones. (3) Undertake habitat restoration and vegetation rehabilitation to improve biodiversity and ecosystem stability. (4) Develop a genetic resource bank and conduct genomic research to monitor and preserve genetic diversity, mitigating the accumulation of deleterious mutations. (5) Implement captive breeding and reintroduction programs to bolster population size and genetic variability. Through implementation and iterative evaluation of these conservation measures, and continuously evaluating and adjusting our strategies, we can hope to reverse the decline of musk deer populations and ensure their long‐term survival in the wild.

## 5 Conclusion

This study provides a comprehensive genomic assessment of endangered *Moschus* species, aiming to elucidate the taxonomic status of *M. anhuiensis* and investigate the genetic distinctions and evolutionary trajectories between *M. anhuiensis* and *M. berezovskii*, alongside their conservation implications. Employing population genetics methodologies, we analyzed whole‐genome re‐sequencing data from both species. Our findings reveal that *M. anhuiensis* is an independent species, which diverged from *M. berezovskii* ~260 kya. The two species have experienced divergent population dynamics, with both populations suffering declines due to anthropogenic activities. *M. anhuiensis* has low genetic diversity, elevated inbreeding levels, and a substantial burden of deleterious mutations. Nonetheless, we identified genes linked to environmental adaptation in the Dabie Mountains. Our findings, by resolving previous taxonomic uncertainties, enhance our understanding of musk deer evolution, the genetic basis of their endangerment, and inform targeted conservation planning.

## Ethics Statement

All animal specimens were approved by the Anhui University Experimental Animal Ethics and Management Committee (IACUC (AHU)‐2024‐040).

## Conflicts of Interest

The authors declare no conflicts of interest.

## Supporting information


Appendix S1.

## Data Availability

The raw sequence data reported in this paper have been deposited in the Genome Sequence Archive, National Genomics Data Center, China National Center for Bioinformation/Beijing Institute of Genomics, Chinese Academy of Sciences (BioProjects RJCA029880), and are publicly accessible at https://ngdc.cncb.ac.cn/gsa.
