# Supplementary material for: Population Genomic Analysis Provides Insights Into the Evolution and Conservation of Two Critically Endangered Musk Deer Species
Source: Evol Appl. 2025 Jul 31;18(8):e70134. doi: 10.1111/eva.70134 (PMC12311986; doi:10.1111/eva.70134)
Supplement: Supplementary file 1 — Appendix S1. [file EVA-18-e70134-s001.docx]

| **Table S1. The sample information of three Musk Deer species individuals** | | | | | | | | |
| --- | --- | --- | --- | --- | --- | --- | --- | --- |
| **Sample** | **Population** | | **Sample** | **Sampling site** | | **species** | **source** | **SRA accession number** |
| DB-1 | | WDB | Tissue | | the wild from the west of Dabie mountain | *M. anhuiensis* | This study |  |
| DB-3 | | WDB | Tissue | | the wild from the west of Dabie mountain | *M. anhuiensis* | This study |  |
| DB-4 | | WDB | Tissue | | the wild from the west of Dabie mountain | *M. anhuiensis* | This study |  |
| DB-5 | | WDB | Tissue | | the wild from the west of Dabie mountain | *M. anhuiensis* | This study |  |
| DB-6 | | WDB | Tissue | | the wild from the west of Dabie mountain | *M. anhuiensis* | This study |  |
| DB-7 | | WDB | Tissue | | the wild from the west of Dabie mountain | *M. anhuiensis* | This study |  |
| DB-9 | | WDB | Tissue | | the wild from the west of Dabie mountain | *M. anhuiensis* | This study |  |
| DB-10 | | WDB | Tissue | | the wild from the west of Dabie mountain | *M. anhuiensis* | This study |  |
| EQL-1 | | EQL | Blood | | ex situ center in Meixian, Shaanxi Province | *M. berezovskii* | Liu et al. 2022 | SRR19913423 |
| EQL-2 | | EQL | Blood | | ex situ center in Meixian, Shaanxi Province | *M. berezovskii* | Liu et al. 2022 | SRR19913422 |
| EQL-3 | | EQL | Blood | | ex situ center in Meixian, Shaanxi Province | *M. berezovskii* | Liu et al. 2022 | SRR19913421 |
| EQL-4 | | EQL | Blood | | ex situ center in Meixian, Shaanxi Province | *M. berezovskii* | Liu et al. 2022 | SRR19913420 |
| EQL-5 | | EQL | Blood | | ex situ center in Meixian, Shaanxi Province | *M. berezovskii* | Liu et al. 2022 | SRR19913419 |
| WQL-1 | | WQL | Blood | | ex situ center in Fengxian, Shaanxi Province | *M. berezovskii* | Liu et al. 2022 | SRR19913418 |
| WQL-2 | | WQL | Blood | | ex situ center in Fengxian, Shaanxi Province | *M. berezovskii* | Liu et al. 2022 | SRR19913417 |
| WQL-3 | | WQL | Blood | | ex situ center in Fengxian, Shaanxi Province | *M. berezovskii* | Liu et al. 2022 | SRR19913416 |
| WQL-4 | | WQL | Blood | | ex situ center in Fengxian, Shaanxi Province | *M. berezovskii* | Liu et al. 2022 | SRR19913425 |
| WQL-5 | | WQL | Blood | | ex situ center in Fengxian, Shaanxi Province | *M. berezovskii* | Liu et al. 2022 | SRR19913424 |
| WSC-1 | | WSC | Tissue | | the wild from the west of Sichuan | *M. berezovskii* | Liu et al. 2022 | SRR16002985 |
| WSC-2 | | WSC | Tissue | | the wild from the west of Sichuan | *M. berezovskii* | Liu et al. 2022 | SRR16002984 |
| WSC-3 | | WSC | Tissue | | the wild from the west of Sichuan | *M. berezovskii* | Liu et al. 2022 | SRR16002983 |
| WSC-4 | | WSC | Tissue | | the wild from the west of Sichuan | *M. berezovskii* | Liu et al. 2022 | SRR16002982 |
| WSC-5 | | WSC | Tissue | | the wild from the west of Sichuan | *M. berezovskii* | Liu et al. 2022 | SRR16002981 |
| DES | | Outgroup | Blood | | Mongolia:Gachuurt Village, Khentii Aimag | *M. moschiferu* | Yi et al. 2020 | [SRR10204416](https://trace.ncbi.nlm.nih.gov/Traces/sra?run=SRR10204416) |

**Table S2. Genome heterozygosity of two musk deer compared to those of other endangered species.**

| Species | Heterozygosity(%) | Source |
| --- | --- | --- |
| Wild horse (*Equus ferus przewalski*i) | 0.052 | Huang et al. 2014 |
| Amur tiger (*Panthera tigris altaica*) | 0.049 | Cho et al. 2013 |
| San Nicolis Island fox (*Urocyon littoralis*) | 0.049 | Robinson et al. 2016 |
| White lion (*Panthera leo*) | 0.048 | Cho et al. 2013 |
| Golden snub-nosed monkey (*Rhinopithecus roxellana*) | 0.042 | Zhou et al. 2016 |
| Bengal tiger (*Panthera tigris tigris*) | 0.040 | Dobrynin et al. 2015 |
| Black snub-nosed monkey (*Rhinopithecus bieti*) | 0.033 | Zhou et al. 2016 |
| San Miguel Island fox (*Urocyon littoralis*) | 0.033 | Robinson et al. 2016 |
| Tasmanian devil (*Sarcophilus harrisii*) | 0.032 | Cho et al. Nat. 2013 |
| Forest musk deer (*Moschus berezovskii*) | 0.031 | this study |
| Siberian tiger (*Panthera tigris altaica*) | 0.030 | Dobrynin et al. 2015 |
| Iberian lynx (*Lynx pardinus*) | 0.010 | Abascal et al. 2016 |
| Yangtze river dolphin (*Lipotes vexillifer*) | 0.026 | Zhou et al. 2013 |
| Snow leopard (*Panthera uncia syn*) | 0.023 | Cho et al. 2013 |
| Cheetah (*Acinonyx jubatus*) | 0.020 | Dobrynin et al. 2015 |
| myanmar snub-nosed monkey (*Rhinopithecus strykeri*) | 0.015 | Zhou et al. 2016 |
| Anhui musk deer (*Moschus anhuiensis*) | 0.013 | this study |
| Baiji (*Lipotes vexillifer*) | 0.012 | Zhou et al. 2013 |
| Iberian lynx (*Lynx pardinus*) | 0.010 | Abascal et al. 2016 |
| Siberian musk deer (*Moschus moschiferus*) | 0.009 | this study |

**Note:** Data from the supplementary file in Hu JY, Hao ZQ, Frantz L, Wu SF, Chen W, Jiang YF, Wu H, Kuang WM, Li H, Zhang YP et al: Genomic consequences of population decline in critically endangered pangolins and their demographic histories. Natl Sci Rev 2020, 7(4):798-814.

**Table S3. 32 genes identified by genome-wide selective sweep (XPEHH and XPCLR)**

| Gene name | Description |
| --- | --- |
| *ROBO1* | roundabout guidance receptor 1 [Source:HGNC Symbol;Acc:HGNC:10249] |
| *PLA2G2C* | phospholipase A2 group IIC [Source:HGNC Symbol;Acc:HGNC:9032] |
| *UBXN10* | UBX domain protein 10 [Source:HGNC Symbol;Acc:HGNC:26354] |
| *CAMK2N1* | calcium/calmodulin dependent protein kinase II inhibitor 1 [Source:HGNC Symbol;Acc:HGNC:24190] |
| *MUL1* | mitochondrial E3 ubiquitin protein ligase 1 [Source:HGNC Symbol;Acc:HGNC:25762] |
| *OLF3* | Olfactory Receptor Family 2 Subfamily F Member 1 |
| *OR6B1* | olfactory receptor family 6 subfamily B member 1 [Source:HGNC Symbol;Acc:HGNC:8354] |
| *IFI203* | None |
| *PDGFRA* | platelet derived growth factor receptor alpha [Source:HGNC Symbol;Acc:HGNC:8803] |
| *SAMM50* | SAMM50 sorting and assembly machinery component [Source:HGNC Symbol;Acc:HGNC:24276] |
| *TTLL12* | tubulin tyrosine ligase like 12 [Source:HGNC Symbol;Acc:HGNC:28974] |
| *TSPO* | translocator protein [Source:HGNC Symbol;Acc:HGNC:1158] |
| *MCAT* | malonyl-CoA-acyl carrier protein transacylase [Source:HGNC Symbol;Acc:HGNC:29622] |
| *TEK* | TEK receptor tyrosine kinase [Source:HGNC Symbol;Acc:HGNC:11724] |
| *SYTL3* | synaptotagmin like 3 [Source:HGNC Symbol;Acc:HGNC:15587] |
| *MTUS2* | microtubule associated scaffold protein 2 [Source:HGNC Symbol;Acc:HGNC:20595] |
| *AJAP1* | adherens junctions associated protein 1 [Source:HGNC Symbol;Acc:HGNC:30801] |
| *PPP2R5C* | protein phosphatase 2 regulatory subunit B'gamma [Source:HGNC Symbol;Acc:HGNC:9311] |
| *RPH3AL* | rabphilin 3A like (without C2 domains) [Source:HGNC Symbol;Acc:HGNC:10296] |
| *CD300H* | CD300H molecule (gene/pseudogene) [Source:HGNC Symbol;Acc:HGNC:52292] |
| *CD300C* | None |
| *CD300LB* | CD300 molecule like family member b [Source:HGNC Symbol;Acc:HGNC:30811] |
| *CDH13* | cadherin 13 [Source:HGNC Symbol;Acc:HGNC:1753] |
| *ATXN1* | ataxin 1 [Source:HGNC Symbol;Acc:HGNC:10548] |
| *GMPR* | guanosine monophosphate reductase [Source:HGNC Symbol;Acc:HGNC:4376] |
| *LSM5* | LSM5 Homolog, U6 Small Nuclear RNA And MRNA Degradation Associated |
| *FSCN1* | fascin actin-bundling protein 1 [Source:HGNC Symbol;Acc:HGNC:11148] |

**Table S4. Genes functional enrichment analysis of 32 Genes**

| Term_id | Term_name | p | Term  size | Size | Intersections |
| --- | --- | --- | --- | --- | --- |
| GO:0005902 | microvillus | 0.002 | 49 | 3 | PDGFRA,TEK,FSCN1 |
| GO:0032101 | regulation of response to external stimulus | 0.0025 | 711 | 7 | ROBO1,CAMK2N1,MUL1,PDGFRA,TTLL12,AJAP1,CDH13 |
| GO:0032102 | negative regulation of response to external stimulus | 0.0028 | 249 | 5 | ROBO1,MUL1,PDGFRA,TTLL12,AJAP1 |
| GO:0042802 | identical protein binding | 0.006 | 1533 | 8 | ROBO1,MUL1,PDGFRA,TEK,MTUS2,CD300LB,CDH13,ATXN1 |
| GO:0009986 | cell surface | 0.006 | 453 | 5 | ROBO1,PDGFRA,TEK,AJAP1,CDH13 |
| GO:0001960 | negative regulation of cytokine-mediated signaling pathway | 0.013 | 50 | 3 | ROBO1,MUL1,TTLL12 |
| GO:0060761 | negative regulation of response to cytokine stimulus | 0.014 | 52 | 3 | ROBO1,MUL1,TTLL12 |
| GO:0098858 | actin-based cell projection | 0.019 | 114 | 3 | PDGFRA,TEK,FSCN1 |
| GO:0005515 | protein binding | 0.035 | 7022 | 15 | ROBO1,UBXN10,MUL1,PDGFRA,TTLL12,TSPO,TEK,SYTL3,  MTUS2,AJAP1,RPH3AL,CD300LB,CDH13,ATXN1,FSCN1 |
| GO:0007266 | Rho protein signal transduction | 0.048 | 78 | 3 | ROBO1,TEK,CDH13 |
| GO:0120025 | plasma membrane bounded cell projection | 0.049 | 1100 | 6 | UBXN10,MUL1,PDGFRA,TEK,CDH13,FSCN1 |


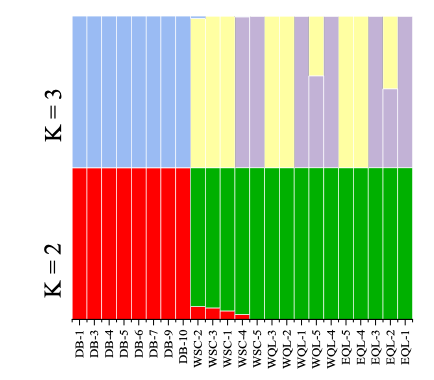


Figure S1 Admixture analysis for *M. anhuiensis* (n = 8) and *M. berezovskii*（n = 15）


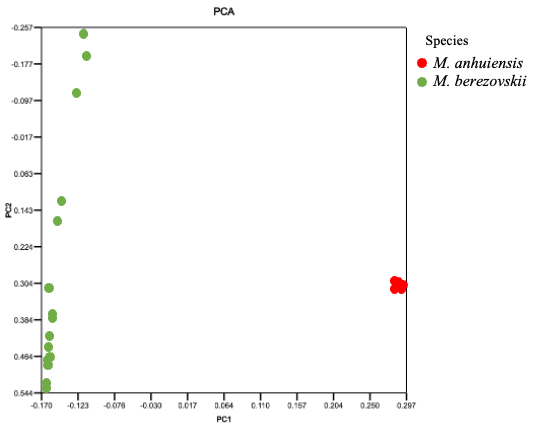


Figure S2 PCA analysis for *M. anhuiensis* (n = 8) and *M. berezovskii*（n = 15）


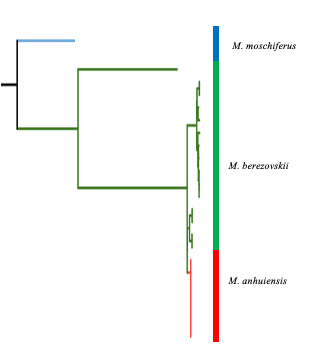


Figure S3. Neighbor-Joining tree base on mtDNA


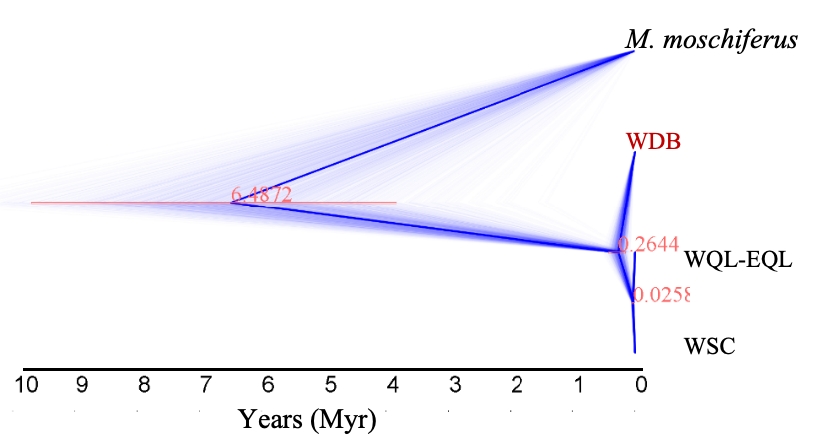


Figure S4. Species/population divergence time analysis using SNAPP, with *M. moschiferus* as the outgroup, and *M. anhuiensis* (WDB) and *M. berezovskii* (WQL-EQL, WSC). All posterior probabilities are greater than 0.9. The median age is indicated above each node, accompanied by the 95% confidence interval. Error bars representing the 95% highest posterior density (HPD) are also shown at the nodes.
